# Citizen approval of nudging interventions promoting healthy eating: the role of intrusiveness and trustworthiness

**DOI:** 10.1186/s12889-018-6097-y

**Published:** 2018-10-19

**Authors:** C. Evers, D. R. Marchiori, A. F. Junghans, J. Cremers, D. T. D. De Ridder

**Affiliations:** 1 Department of Social, Health, and Organizational Psychology, Utrecht University, P.O. Box 80140, 3508 TC Utrecht, The Netherlands; 2 Department of Methodology and Statistics, Utrecht University, Utrecht, The Netherlands

**Keywords:** Nudging, Citizen opinions, Health policy, Eating behavior, Choice architect

## Abstract

**Background:**

Nudging interventions have lately been widely adopted by policy makers to increase the welfare of society and to help citizens make better choices. Hence, it has become important to understand the conditions under which they are approved. While most research has looked into whether professionals approve of nudging interventions, surprisingly the opinion of the target group has been widely ignored. This study investigated citizens’ level of approval of nudging in the realm of healthy eating promotion, as well as its boundary conditions.

**Methods:**

Participants (*N* = 1441) from the US and seven European countries were probed for their level of approval of nudges. Moreover, we investigated whether these levels of approval were dependent on the level of intrusiveness of the nudge and on the type and trustworthiness of the source (policy makers, experts, industry) implementing the nudge.

**Results:**

People revealed moderate to high levels of approval with nudging across all countries. Intrusiveness and nudging approval were negatively associated. Nudges implemented by experts received more approval than those by policy makers. In general, approval increased with the trustworthiness of the source.

**Conclusions:**

These results provide information for European and American policy makers considering using nudging in their policy repertoire.

## Background

Many people are aware of the techniques that companies use to influence consumers to buy their products or services. Food products placed near the cash register, star ratings and reviews from unknown satisfied customers, or pre-selected options to make us adhere to their recommendations. When companies employ such techniques to increase their sales, we speak of marketing. However, when governments use these techniques for the benefit of people, we speak of nudging. Nudging (implementing choice architecture) is a strategy that aims at influencing people’s behaviors in predictable ways without excluding any options from the choice array or meaningfully changing the costs involved in any option [1]. Banning junk food or raising its price is not a nudge, but putting fruits at eye level is. While these interventions have been used for decades by private companies to increase their own benefit, recently policy makers have become interested in implementing nudging in policy with the purpose of facilitating desired choices [2]. The purpose of nudging is to influence people in various life domains (e.g., recycling, energy conservation, organ donation) in order to increase the welfare of society and to encourage citizens in making better choices.

In doing so, nudges respect that people often do not deliberate much on their decisions but are most of the time inclined to use swift, intuitive or habitual ways of thinking (also addressed as so-called System 1 thinking as opposed to slow and rational System 2 thinking [3]). In contrast to traditional ways of behavior change interventions that are often used in public health, nudges do not aim to educate or persuade people to change their behavior but rather speak to their mindless way of making choices that relate to health behavior [4]. Some scholars have raised questions about the ethics of nudging, suggesting that nudges may harm autonomous decision making and that people are being manipulated by nudges that are beyond their awareness [5]. Others, however, have argued that nudges do respect autonomy because they support people in doing what they want but often forget in the spur of the moment [6].

Linked to these contrasting views, policy makers experience difficulties in implementing nudges as they lack insights into people’s attitudes towards nudging [7–11]. Indeed, nudging by policy makers poses the question to what extent people can be influenced in their everyday life. Which behaviors are off limits? Which nudging interventions are most effective? Who is most accredited to implement them?

While most research looked into how professionals (e.g., lawyers, ethicists, policy makers) think about nudges, surprisingly, the feedback of the target group has been widely ignored. Research is thus needed into citizens’ attitudes and conditions for approval with nudging to alleviate some policy makers’ remaining reluctance [12]. The aim of this paper is to fill this gap and to investigate the boundary conditions of nudging interventions and how they are received by the target population, the citizens themselves. The results from this study will provide information for policy makers considering using nudging in their policy repertoire.

Recently, large surveys conducted in Europe, the Americas, Africa, Asia and Australia showed that nudging may be perceived differently depending on the country of residence [13, 14]. These studies showed that European countries such as Hungary and Denmark, and other countries such as Japan and Canada reported relatively low scores of approval compared to other countries such as Italy, France, China, and South Korea. The authors of these studies postulated that these differences may be due to people’s different evaluations of the demand levels (e.g., financial cost, time) that nudges will have on their life, the legitimate ends of the nudging interventions, and whether they are consistent with their personal interests and values. Two main aspects that could then explain these differences are intrusiveness and trustworthiness. Intrusiveness relates to the extent to which the nudges complement or disrupt people’s daily life routines and goals, thus how intrusive people regard the nudges to be. Trustworthiness relates to the extent to which people can trust the choice architect’s decisions to uphold and promote their interests and values with the implemented nudges (i.e., the source or the person implementing the nudge). In other words, both intrusiveness an trustworthiness seem to predict nudging approval, with intrusiveness relating to the nudge intervention and trustworthiness relating to the source of the nudge intervention.

To elaborate on intrusiveness firstly, recent research showed that people can display a degree of psychological reactance to being nudged upon learning that they have been influenced [15]. This suggests that one cause for disapproval is not that someone objects to the promoted behavior itself, but to the mere fact that it is externally induced rather than instigated by the person himself. The differences in approval of nudges has also been suggested to result from the level of intrusiveness of the nudges in people’s daily routines and life [14]. Non-intrusive nudges, such as providing information on calorie content, found more approval than more intrusive nudges, such as defaults (providing a preselected option) [13, 14]. In these studies, the researchers decided beforehand to categorize the nudges according to different levels of intrusiveness. In order to further test this relationship, however, it seems important to let participants themselves rate the level of intrusiveness of the different nudges.

The second aspect that may moderate nudging approval, trustworthiness of the source, is related to the current debate on whether governments should be allowed to implement nudging interventions. Indeed, researchers have suggested that low levels of approval of nudging in countries such as Hungary or Japan may be due to the level of trust they attribute to their governments [14]. Nudges that were supposedly promoted or mandated by the government received the least approval. Junghans and colleagues [16] also observed that citizens’ approval was contingent upon the source of the nudge. Nudges originating from trusted sources with good intentions such as experts in the specific nudged behavioral domain were more approved. The moderating role of the source was further supported in a recent study showing that people approve nudges depending on whether the source proposing the nudge matches their political orientation [17]. Hence, next to the intrusiveness of the nudge, another important factor in the approval of nudging is the source implementing the intervention. The extent to which both factors relate to the approval of nudges will be investigated in the current research.

### Current research

The goal of the current study was to investigate citizens’ level of approval of nudging. Past research found that people’s approval of nudges depends on the behavioral domain in which the nudge was implemented [12]. As the type of behavior that is nudged is thus likely to be an important moderator, we deemed it important to focus on one prototypical behavior: healthy eating. For several reasons we chose for healthy eating. First, people have to engage in eating several times a day. In our obesogenic environment people do not only have to decide how much they eat, but also what they eat. Accordingly, not only people themselves, but several important stakeholders are involved in the choice architecture to which people are exposed to, ranging from industry to policy makers. Research has revealed that people generally hold positive attitudes towards nudging strategies in the realm of health behavior [14, 16, 18]. This may be related to many people having a goal to maintain a healthy diet and being aware of the impact of healthy eating on their health and well-being [16, 19, 20].

Further in order to provide a more comprehensive picture on the boundary conditions of citizens’ opinions and approval of nudges, we decided to investigate people’s opinions in the USA and in several European countries with different historical, geographical, and cultural backgrounds to increase the generalizability of the findings. Moreover, based on previous findings that showed that some socio-demographic variables may also predict nudging approval [14, 17], we assessed and analyzed in an exploratory capacity, whether gender, age, Body Mass Index (BMI), and political orientation (liberalism vs. conservatism) would impact approval of nudges promoting healthy eating behaviors.

We assumed that approval would be different depending on the type of nudge and on who nudged (the source). Related to the type of nudge, each participant was provided with three different nudge types that had to be scored on intrusiveness. We hypothesized that different nudge types would result in different approval scores (*Hypothesis 1a*) and the relation between type of nudge on approval to be mediated by the intrusiveness of the nudge *(Hypothesis 1b)*; nudge interventions perceived as less intrusive were assumed to receive more approval. Related to the source, the nudge interventions were chosen to come from three different sources that participants had to score on trustworthiness. These sources were policy makers, experts, and those in industry. Based on previous research, we hypothesized that approval would be highest for experts and lowest for policy makers (*Hypothesis 2a*). Furthermore, we hypothesized that the relation between source and approval to be mediated by the trustworthiness of the source (*Hypothesis 2b*); sources perceived as more trusty were assumed to receive more approval. Thus, we assumed two different mediation models. On the one hand we expect the relation between type of nudge on approval to be mediated by intrusiveness. On the other hand we expect the relation between type of source on approval to be mediated by trust.

## Methods

### Participants

We recruited 1475 participants in eight countries using the crowdsourcing platforms Amazon Mechanical Turk (for the US) and Crowdflower for European countries (Germany, the Netherlands, France, Italy, Poland, Bulgaria, and the UK). We randomly chose a diverse set of different countries across Europa and the US to gain a variety of different Western nations. Further, Amazon Mechanical Turk and Crowdflower are online marketplaces commonly used for social science research due to its provision of easy access to a broad sample of the general public that allows for better insights than the commonly used university student samples [21]. Nevertheless, selection biases with internet platforms may occur, particularly considering differences in samples across different countries. For that reason, we refrained from statistically comparing the results from samples of different countries and merely provide descriptive data.

In total 32 participants were excluded from the analysis for not providing all required information and two for reporting to be younger than 16 years of age, resulting in a sample of 1441 participants. They were on average 35.35 years old (*S.D.* = 10.77) with a BMI of 24.67 (*S.D.* = 5.98), and 533 (37%) were female participants. Sample sizes varied between countries due to different participation rates in the survey (see Table 1).

**Table 1:** Sample size for each country by Gender, and Age and BMI (data are mean scores (SD))

Country	N		Age	BMI
Germany	Male	156	38.2 (12.86)	25.36 (4.88)
	Female	46		
	Total	202		
UK	Male	79	37.5 (10.82)	24.95 (5.97)
	Female	77		
	Total	156		
France	Male	140	33.84 (10.61)	23.93 (4.98)
	Female	59		
	Total	199		
Poland	Male	94	33.24 (10.03)	24.43 (4.38)
	Female	34		
	Total	128		
USA	Male	83	35.98 (10.87)	28.38 (11.18)
	Female	54		
	Total	137		
Bulgaria	Male	200	35.39 (9.69)	23.52 (3.89)
	Female	157		
	Total	357		
Netherlands	Male	60	35.96 (11.84)	26.58 (8.41)
	Female	23		
	Total	83		
Italy	Male	96	32.57 (9.09)	23.2 (3.64)
	Female	83		
	Total	179		

Omnibus ANOVAs comparing age, *F*(7, 1433) = 6.12, *p* < 0.001, and BMI, *F*(7, 1433) = 13.88, *p* < 0.001, of the different countries show significant differences. For age, post-hoc analyses with Bonferroni corrections showed that Germany differed significantly from France (*p* = 0.001), Poland (*p* = 0.001) and Italy *(p* < 0.001). The UK also differed significantly from France (*p* = 0.038), Poland (*p* = 0.023) and Italy (*p* = 0.001). For BMI, post-hoc analyses showed that the USA differed significantly from all other countries (*p* < 0.001) except the Netherlands. The Netherlands differed significantly from France (*p* = 0.013), Bulgaria (*p* < 0.001) and Italy (*p* < 0.001). Finally, Germany differed significantly from Bulgaria (*p* = 0.009) and Italy (*p* = 0.009).

### Design and procedure

Questionnaires in the Official National Language of the participating countries were available for participants. Questions were translated from an original English version by researchers who were native speakers, or were back translated to ensure equivalency of meaning. After providing informed consent, participants were introduced to three examples of nudges promoting healthy eating which are common in the literature [22, 23] (without mentioning of the term nudging):

Example 1 “shopping”: *To help people eat healthily, the manner in which healthy and unhealthy foods are presented could be changed. For example: When shopping for food, healthy food could be placed more visibly (at eye level or near the cash register) than unhealthy food to help people resist the temptation of buying unhealthy food. In this example people could still choose any food they like, healthy and /or unhealthy; all options would remain available.*

Example 2 “tableware”: *To help people eat healthier amounts of food, the available tableware could be changed. For example: When getting dinner, smaller plates could be given to people to help them choose smaller portion sizes and eat less. In this example people could still choose any amount of food they like; all options would remain available.*

Example 3 “snacks”: *To help people eat healthily, the manner in which healthy and unhealthy foods are presented could be changed. For example: In situations where people like to snack, healthier snack options could be made more available (for example in vending machines) to help people choose a healthier snack. In this example people could still choose any snack option they like, healthy and /or unhealthy; all options would remain available.*

These examples were drawn from reviewing the recent literature on nudging and the examples provided therein. The selection criteria were set to ensure that examples included different situations in which nudges were applied, different target behaviours, and to embed different nudging strategies.

### Dependent measures

Following each randomly presented example participants were asked “*To what degree would you approve if this measure was implemented by…?*” (1 = Very much disapprove – 7 = Very much approve). This question was posed three times, once for each source, namely *the food industry, policy makers,* and *independent experts in the field*, at random order. Subsequently, each example was repeated in random order without reference to a specific source and participants were asked “*How intrusive do you find this measure?”* (1 = Not at all intrusive – 7 = Very intrusive). The trustworthiness of each source was assessed by asking “*To what degree do you regard [the source] as trustworthy?”* (1 = Not at all trustworthy – 7 = Very trustworthy) in random order. Finally, demographic variables, including age, gender, BMI, political orientation (liberal – conservative), and country of residence were assessed. Finally, participants were debriefed and thanked.

### Analytic strategy

Before each mediation model was fit, separate repeated measures ANOVAs were used to check whether the predictor variable (nudge or source) influenced and the approval scores (outcome), respectively to test *Hypotheses 1a and 2a*. Next, the same statistical analysis was used to test whether the predictor variable influenced the intrusiveness or trust scores (mediator). Contrast analyses were done to see which nudges or sources differed significantly on the outcome and mediators.

Two mediation models were fit to test the mediation of intrusiveness on the effect of type of nudge on approval (*Hypothesis 1b*) and the mediation of trust on the effect of source on approval (*Hypothesis 2b*) using the mediation package in R [24–26]. Because in the models the predictor variable (source or nudge) is categorical, we assessed k-1 (where k = 3 is the number of categories of the predictor) mediation models. In both models the nudge or source with the lowest average approval score was chosen as the reference category. In the package, mediation confidence intervals for the effects are computed by using a quasi-Bayesian Monte Carlo method based on a normal approximation.

For *Hypothesis 1b*, approval scores of each participant were averaged over sources, resulting in three measurements (one for each nudge) per participant for the analysis of the first mediation model. In the second mediation model, i.e., *Hypothesis 2b*, approval scores (i.e., the outcome variable) of each participant were averaged over nudges resulting in three measurements (one for each source) per participant. Participant was the grouping factor in both models. The mediation models thus contain two levels, participant and source of nudge, respectively the between and within participant levels. Next to the outcome variable we measured a mediator at the within participant level. Trust was measured for each source and Intrusiveness was measured for each nudge. The variables intrusiveness and trust were not centred but 0 was included in their scale by subtracting 1 unit (the new range is 0 to 6).

## Results

### Sample descriptive

**Fig. 1:**
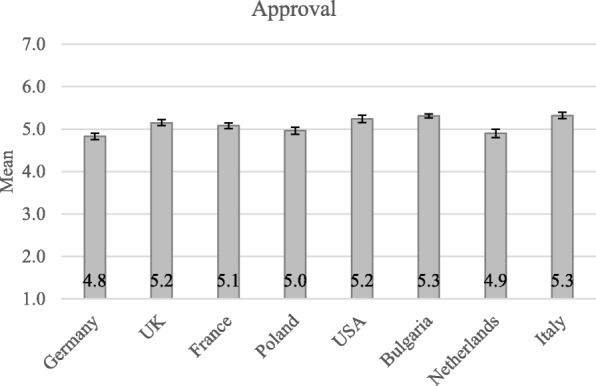
Average Approval by country of residence

Descriptive analyses revealed moderately high degrees of approval for all three nudges promoting healthy eating (*M* = 5.13; *S.D.* = 0.99). This corroborates previous research suggesting that attitudes towards nudging are positive in domains where citizens understand the decision-making context and/or value the outcome behavior. Exploratory ANOVA analysis showed that approval differed per country, *F*(7, 1433) = 6.92, *p* < 0.001. Approval was strongest in Italy (*M* = 5.32, *S.D.* = 0.99) and weakest in Germany (*M* = 4.83, *S.D.* = 1.05). Figure 1 shows how approval slightly differs by country of residence. Post-hoc analyses with Bonferroni corrections showed that Germany differed significantly from USA (*p* = .005), Bulgaria (*p* < .001), and Italy (*p* < .001). Italy also differed significantly from Poland (*p* = .036) and the Netherlands (*p* = .032). Finally, Bulgaria differed significantly from Poland (*p* = .015) and the Netherlands (*p* = .018).

Further exploratory analysis on the effects of socio-demographic variables on approval did not provide evidence for a relationship between political orientation and approval, *r* = −.24, *p* = .81. BMI and age were also not related to approval *r* = −.42, *p* = .11 and *r* = .06, *p* = .83, respectively. Finally, the data revealed an effect of gender, *F*(7, 1439) = 80.48, *p* < .001, with higher approval for nudges in eating behavior by women (*M* = 5.43, *S.D.* = 0.93) than men (*M* = 4.96, *S.D.* = .98). As approval scores were quite similarly positive across countries, we merged all the participants into one big sample for the two main analyses. Including gender and country of residence as covariates in the main analyses did not change the significance nor the pattern of results.

### Effects of the type of nudge and its intrusiveness on approval of nudging

**Fig. 2:**
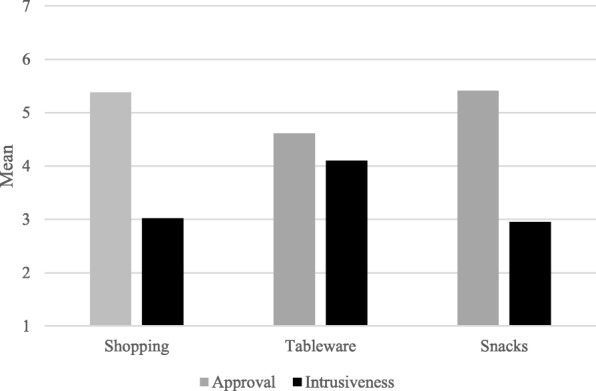
Mean ratings of Approval and Intrusiveness for each nudge across sources

Our first hypothesis stated that the type of nudge would lead to different approval scores (*Hypothesis 1a*), and that this effect may be related to the degree of intrusiveness of the nudging intervention (*Hypothesis 1b*). We expected less intrusive nudges to be more approved of. We first examined whether approval differed for the three different nudges. Results of a repeated measures ANOVA indeed showed that approval scores significantly differed between nudge types, *F*(1, 1440) = 337.93, *p* < .001, confirming *Hypothesis 1a*. As shown in Fig. 2, the second nudge “tableware” promoting a dinner plate change (*M* = 4.61, *S.D.* = 1.40) was significantly less approved than the nudge examples “shopping” promoting healthy food visibility (*M* = 5.38, *S.D.* = 1.19; *F*(1,1440) = 415.17, *p* < .001) and “snacks” promoting healthy snack availability (*M* = 5.41, *S.D.* = 1.15; *F*(1,1440) = 434.74, *p* < .001). Next, we examined the perceived degree of intrusiveness of the nudges. Overall, perceived intrusiveness of the nudges was low to moderate (on a scale of 1 to 7: *M* = 3.36; *S.D.* = 1.52). To examine whether the three nudges differed in perceived intrusiveness, a repeated measures ANOVA was performed. Results revealed that the nudges differed significantly, *F*(1,1440) = 320.51, *p* < .001. As shown in Fig. 2, the “tableware” nudge (*M* = 4.10, *S.D.* = 1.91) was perceived as significantly more intrusive than the nudges relating to “shopping” (*M* = 3.02, *S.D.* = 1.88; *F*(1, 1440) = 372.22, *p* < 0.001) and “snacks” (*M* = 2.95, *S.D.* = 1.87; *F*(1, 1440) = 415.89, *p* < 0.001).

Finally, we analyzed whether the effects of the type of nudge on approval were mediated by the degree of intrusiveness of the nudge (*Hypothesis 1b*). Given that the predictor variable (i.e., type of nudge) had three levels, we choose the nudge with the lowest average approval (i.e., Nudge 2 “tableware”) as the reference category. First, we compared the “tableware” nudge to the “shopping” nudge. The average causal mediated effect (ACME) was 0.22, *p* < 0.001 (see Table 2). Both the average direct effect (ADE = 0.55, *p* < 0.001) and the total effect (0.77, *p* < 0.001) were significant. In other words, the increase in approval from the “tableware” nudge to the “shopping” nudge (i.e., 0.77) is explained for 29% (i.e., 0.22) by the difference in intrusiveness between both nudges. Lastly, we compared the “tableware” nudge to the “snack” nudge. The average causal mediated effect (ACME) was 0.24, *p* < 0.001. Both the average direct effect (ADE = 0.56, *p* < 0.001) and the average total effect (0.80, *p* < 0.001) were significant. This means that the increase in approval from the “tableware” nudge to the “snack” nudge (i.e., 0.80) is explained for 30% (i.e., 0.24) by the difference in intrusiveness between both nudges.

**Table 2:** Average causal mediated effect (ACME) and average direct effect (ADE) and total effect for the mediation model in which the effect of the type of nudge on approval is mediated by the degree of intrusiveness of the nudge

	Nudge “tableware” vs. nudge “shopping”	Nudge “tableware” vs. nudge “snacks”
ACME (% of total)	0.22 (29%)	0.24 (30%)
ADE (% of total)	0.55 (71%)	0.56 (70%)
Total Effect	0.77	0.80

In sum, results show that, as predicted, intrusiveness significantly mediated the effects of the type of nudge on approval ratings of nudges. Figure 2 shows how the lower the participants rated the intrusiveness of a nudging intervention the more they approved of the intervention.

### Effects of the type of source and its trustworthiness on approval of nudging

**Fig. 3:**
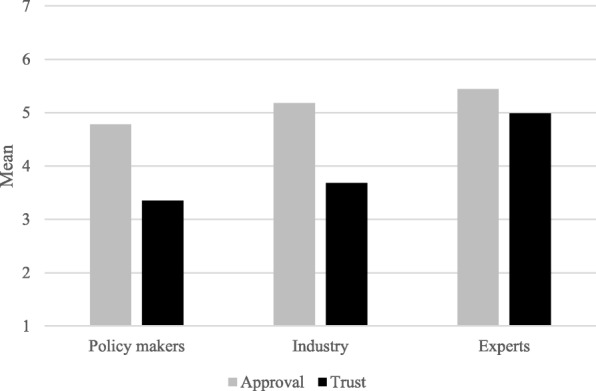
Mean ratings of Approval and Trust for each source across nudges

Our second hypothesis stated that the type of choice architect implementing a nudging intervention would influence people’s attitude towards it (*Hypothesis 2a*), and that this effect might be due to the level of trust in the choice architect (*Hypothesis 2b*). In other words, we expected more trusted sources to be more approved of, with experts being highest and policy makers lowest. In order to do so, we first examined whether the three different sources differed on approval. As expected, results of a repeated measures ANOVA showed that the type of source significantly influenced approval scores, *F*(1, 1440) = 290.26, *p* < 0.001. Figure 3 shows how policy makers (*M* = 4.78, *S.D.* = 1.31) were significantly less approved of than industry (*M* = 5.18, *S.D.* = 1.14; *F*(1, 1440) = 199.79, *p* < 0.001) and experts (*M* = 5.44, *S.D.* = 1.01; *F*(1, 1440) = 492.67, *p* < 0.001). Next, we examined the perceived degree of trustworthiness of the sources. Overall, the perceived trustworthiness of the sources was moderate (on a scale of 1 to 7: *M* = 4.01; *S.D.* = 1.15). Results from a repeated measures ANOVA revealed that trustworthiness differed between the three sources, *F*(1, 1440) = 709.93, *p* < 0.001. As shown in Fig. 3, experts (*M* = 4.99, *S.D.* = 1.41) were perceived to be significantly more trustworthy than policy makers (*M* = 3.35, *S.D.* = 1.58; *F*(1, 1440) = 1335.72, *p* < 0.001) and industry (*M* = 3.68, *S.D.* = 1.61; *F*(1, 1440) = 776.85, *p* < 0.001).

Finally, we analyzed whether the effects of the type of source on approval were mediated by the degree of trustworthiness of the source (*Hypothesis 2b*). Given that the predictor variable (i.e., type of source) had three levels, we choose the source with the lowest average approval (i.e., Policy makers) as reference category. First, we compared policy makers to industry. The average causal mediated effect (ACME) was 0.09, *p* < 0.001 (see Table 3). Both the average direct effect (ADE = 0.31, *p* < 0.001) and the average total effect (0.40, *p* < 0.001) were significant. In other words, the increase in approval from policy makers to industry (i.e., 0.40) is explained for 23% (i.e., 0.09) by the difference in trustworthiness between both sources. Lastly, we compared policy makers to experts. The average causal mediated effect (ACME) was 0.45, *p* < 0.001. Both the average direct effect (ADE = 0.21, *p* < 0.001) and the average total effect (0.66, *p* < 0.001) were significant. This means that the increase in approval from policy makers to industry (i.e., 0.66) is explained for 69% (i.e., 0.45) by the difference in trustworthiness between both sources.

**Table 3:** Average causal mediated effect (ACME) and average direct effect (ADE) and total effect for the mediation model in which the effect of the type of source on approval is mediated by the degree of trustworthiness of the source

	Source: Policy makers vs. Industry	Source: Policy makers vs. Experts
ACME (% of total)	0.09 (23%)	0.45 (69%)
ADE (% of total)	0.31 (77%)	0.21 (31%)
Total Effect	0.40	0.66

In sum, results show that, as predicted, trustworthiness significantly mediated the effects of the type of source on approval ratings of nudges. Figure 3 shows how the higher the participants rated the trustworthiness of a source the more they approved of nudges implemented by that source.

## Discussion

The present study examined the boundary conditions of people’s approval of nudges promoting healthy eating. Results showed moderate to high levels of approval. This finding was confirmed across eight countries with different historical, geographical, and cultural backgrounds. Approval was highly dependent on the degree of perceived intrusiveness of the nudge and on the degree of trust put in the choice architect implementing the intervention. Nudges implemented by experts and industry, as opposed to policy makers, were more approved of. In general, approval was higher when perceived intrusiveness was low and when trust in the choice architect was high. These findings provide information for policy makers considering the implementation of nudging strategies on how to design them for maximum approval and effectiveness.

Notably, consumer opinions about nudging are important to consider in view of the heated debate amongst professionals who have contrasting views on the functionality of nudging as an effective intervention strategy. Whereas many scholars are in favor of using nudges in public health, specifically for the benefit of lower educated people who experience difficulties in processing complex health information [27], it has been suggested that nudges do not account for structural health inequalities and that governments should take responsibility for legislation and other firm measures in public health policy [28].

Importantly, the results of the present study are specific to the realm of eating behavior. This restriction avoided previously observed differences in citizens’ attitudes towards nudging in different behavioral domains [16] and ensured that policy makers can draw direct conclusions for the realm of eating behavior. Future studies could examine whether the present findings extend to other eating related nudges such as accessibility of food, distance, attractiveness, package size or social norms [16–20, 22–26, 29–33].

In relation to future studies, it would also be valuable to learn to what extent people’s approval of nudges relates to acting upon nudges. Forthcoming research could, for example, investigate the present research questions in people who have and have not adjusted their behavior in response to nudging in order to assess if approval initiates more influence on people’s behaviour that is being nudged.

### Intrusiveness

The importance to examine the effects of different nudge types on approval is further confirmed by the fact that approval of nudges was higher when they were perceived as less intrusive. The more intrusive nudge, and thus least approved of, intended to change the size of the plates and bowls in people’s homes. The least intrusive one, and more approved of, suggested to change the range of foods found in shops or vending machines. In other words, the less a nudge would disrupt people’s daily lives or ask them to make personal changes, the more accepted it was. This attitude might be explained by a strong reactance to change personally in contrast to external changes. These findings also build on previous studies that showed that nudges which just displayed additional information, such as calorie content, were more approved of than nudges which aimed to make a pre-selection in a person’s stead, such as defaults [13, 14]. These latter studies presented preliminary evidence of how the degree of intrusiveness of a nudge might have important consequences on its approval and probably effectiveness, which were confirmed in the present study. Policy makers, but also practitioners in general, aiming to implement nudges should thus take into account how much disruption a nudging intervention might have on people’s lives and daily routines. However, given that the least intrusive nudges are considered the most acceptable by the target populations may also be less effective, policy makers should consider striking a balance between intrusiveness and effectiveness of nudges.

### Trustworthiness

Next to the degree of intrusiveness of a nudge, results showed that the degree to which a source is trusted strongly impacted people’s approval with nudging. Specifically, when a nudge was said to be implemented by policy makers, participants disapproved more of the intervention than when it was said to be implemented by the industry or independent experts. These results confirm previous research that people are usually skeptical about governmental interventions [16]. Our findings showed that this differentiated level of approval depending on the source implementing a nudge was highly contingent on the degree to which the source was trusted. Consistently, previous research in the fields of communication and political science has shown that messages promoted by trusted sources are typically more accepted and acted upon [34–36]. Hence, countries that display high levels of trust towards their policy makers should not fear to use nudges to improve people’s lives and social welfare. Otherwise, these findings suggest that policy makers and practitioners implementing nudges should involve experts in the target behavioral domain during the design of these nudges and during the decision about which behaviors and choices ought to be promoted. Future research should however examine whether these differences in approval are mostly due to a general distrust in governments or to a general acceptance that industry is meant to market their products in any way they want [14]. The finding that (local) governments are least trusted by the target audience may be not specific for nudge policies. Yet, given that (local) governments are generally responsible for implementing nudges is worrying and indicates that nudges policies need careful explanation.

### Country and gender differences

In contrast to previous research showing no gender differences in approval [15], our data revealed that women were more approving of nudges than men. This effect could be driven by the fact that eating, body size, and image are often a stronger concern for women than men and that the preparation of food is a traditionally female role, which could drive the stronger approval of nudges supporting healthy eating for women [37, 38]. Moreover, it has been speculated that women are in general more empathetic and more interested in the fate of other persons than men [39] which would lead them to be more supportive of health and safety nudges.

The correspondence of results across countries, especially between the US and European countries, stands in contrast to the reasoning by Sibony and Alemanno [40] that Europeans should be more “intervention-friendly” (p. 23) than the US due to differences in their legal cultures. However, although approval with nudging was generally high, there were some differences between countries. Specifically, approval was strongest in Italy and Bulgaria, and weakest in Germany and the Netherlands. However, these results should be carefully interpreted for several reasons. First, the correlational nature of the data does not allow any causal inferences. Secondly, while the data collected in each country are informative for the specific country itself, direct comparisons are not allowed due to the non-representative sampling. For example, only 37% of the participants was female and there were differences across countries in age and BMI. Future studies should investigate additional differences between countries and what causal factor drives these potential differences.

## Conclusions

Nudging is an inexpensive and effective strategy to influence people’s decisions and society’s welfare. The increased interest by policy makers in the use of these interventions has raised questions regarding the limits of application and the boundary conditions to success and approval. The present study offers reassurance regarding citizens’ approval to implement these interventions in their daily life. It highlighted however the necessity to 1) use trusted sources to design and implement nudges that 2) do not intrude too much in people’s daily lives and routines. In conclusion, this study makes a theoretical advance by revealing the conditions for people’s approval with nudging healthy eating behavior and provides practical information for policy makers considering the inclusion of nudging in their policy repertoire in a number of European countries and the US.

## Abbreviations

ACME: Average Causal Mediated Effect
ADE: Average Direct Effect
BMI: Body Mass Index
